# ﻿Two new species of *Helochares*, with additional faunistic records from China (Coleoptera, Hydrophilidae, Acidocerinae)

**DOI:** 10.3897/zookeys.1078.73458

**Published:** 2021-12-16

**Authors:** Zhenming Yang, Fenglong Jia, Yudan Tang, Lu Jiang

**Affiliations:** 1 Institute of Entomology, Life Science School, Sun Yat-sen University, Guangzhou, 510275, Guangdong, China Sun Yat-sen University Guangzhou China; 2 Shenzhen Mingde Experimental School, Shenzhen, Guangdong, China Shenzhen Mingde Experimental School Shenzhen China; 3 Shenzhen Wildlife Conservation Division, Shenzhen, Guangdong, China Shenzhen Wildlife Conservation Division Shenzhen China

**Keywords:** New records, Oriental Realm, species distribution, taxonomy, water scavenger beetle

## Abstract

Two new species, *Helocharesguoi* Yang & Jia, **sp. nov.** and *Helocharesdistinctus* Jia & Tang, **sp. nov.**, are described. Two species are recorded for the first time from China: *Helocharesnegatus* Hebauer, 1995 from Yunnan, and *Helocharesminusculus* d’Orchymont, 1943 from Guangdong. Additional faunistic data from China are provided for the following species: *Helochareshainanensis* Dong & Bian, 2021, *Helocharesnipponicus* Hebauer, 1995, *Helocharessauteri* d’Orchymont, 1943, *Helocharesdensus* Sharp, 1890, *Helochareslentus* Sharp, 1890, *Helocharesneglectus* (Hope, 1854) and *Helocharesanchoralis* Sharp, 1890. The Chinese fauna of *Helochares* comprises 16 species, 11 of which are illustrated in this contribution. *Helocharescrenatus* Régimbart, 1921 is removed from the Chinese fauna.

## ﻿Introduction

*Helochares* Mulsant, 1844 is one of the most diverse and widespread genera of Hydrophilidae, mainly distributed in the Afrotropical, Oriental and Australian realms, with a few species also present in the Palearctic, Neotropical and Nearctic realms. d’Orchymont (1919) recognized five subgenera within *Helochares*: *Hydrobaticus* MacLeay, 1871, *Chasmogenus* Sharp, 1882, *Helochares* Mulsan, 1844, *Helocharimorphus* Kuwert, 1890 and *Sindolus* Sharp, 1882. Hansen (1991) added Batochares Hansen, 1991 as a subgenus of Helochares although he recognized *Helochares* as a polyphyletic group at that time. Fernández (1986) separated subgenus Chasmogenus from *Helochares* and reinstated its generic status. Subgenera *Batochares* and *Sindolus* were elevated to generic status based on the molecular phylogeny by Short et al. (2021). The remaining three subgenera, *Helochares* (s. str.), *Hydrobacticus* and *Helocharimorphus* were synonymized with *Helochares* based on molecular phylogeny and morphological characters (Girón and Short 2021; Short et al. 2021).

So far, 159 species have been described worldwide (Hansen 1999; Short and Hebauer 2006; Short and Fikáček 2011; Girón et al. 2021), but there is no detailed revision of any continent although many species have been described since the end of the last century (e.g., Hebauer 1995, 1998, 2002; Matsui 1995; Hebauer et al. 1999) except for the revision of subgenus “*Hydrobaticus*” of the New World (Short and Girón 2018).

The fauna of Chinese *Helochares* is poorly known. The first Chinese species, *H.neglectus*, was described by Hope (1845) from Guangzhou, Guangdong Province. Since then, 16 species have been recorded (e.g., d’Orchymont 1919, 1925, 1940, 1943a, b; Pu 1963; Gentili et al. 1995; Fikáček et al. 2015; Jia and Tang 2018; Dong and Bian 2021). Of all known Chinese species, thirteen occur south of the Yangtze River, and the other three in the northwest, northeast and southwest China (Jia and Tang, 2018; Dong and Bian 2021). Adult *Helochares* (s. str.) usually occur in ponds and at the edge of slow streams, or on surface of wet stones covered with leaves (*H.fuliginosus* d’Orchymont). In China, *Helochares* is the only hydrophilid genus in which adult females carry their egg cases beneath their abdomens.

## ﻿Material and methods

Male genitalia were dissected in some specimens of each species. Dissected genitalia were transferred to a drop of absolute alcohol for removing membranes after 8–10 hours in 10% KOH at room temperature, and subsequently mounted in a drop of glycerine on a piece of transparent plastic slide attached below the respective specimens. Morphological characters of the male genitalia were examined using a Nikon SMZ800 compound microscope. Genitalia photographs were taken using a Zeiss Axioskop 40 compound microscopes and combined with AutoMontage software version 3.8. Photographs of habitus and external morphology were taken using a Leica M205C stereomicroscope and combined with AutoMontage software.

Detailed descriptions of *Helochares* were provided by Hansen (1991). Morphological terminology largely follows Hansen (1991) and Komarek (2004).

Examined specimens are deposited in the following collections:

**IRSN**Institute Royal de Sciences naturelles, Brussels, Belgium;

**IZCAS**Chinese Academy of Sciences, Institute of Zoology, Beijing, China;

**SYSU**Entomological Collection of Sun Yat-sen University, Guangzhou, China.

Specimens in which the depository is not indicated are deposited in SYSU.

## ﻿Taxonomy

### ﻿ Helochares guoi

Taxon classificationAnimaliaColeopteraHydrophilidae

Yang & Jia sp. nov.

B0CCAC74-9271-5E48-89D9-9262DC3CF04E

http://zoobank.org/2BAF353A-6A2D-439C-BBF0-08A84906B3E4

Figs 1–2 , 6 , 8–9 , 24–26

#### Material examined.

***Holotype***: Male, Guangdong, Shenzhen, Dapeng Peninsula, Getian village, 22.48175°N, 114.52643°E, 2.viii.2019, Fenglong Jia and Zuqi Mai leg. ***Paratype***: 1 female, same data as holotype.

#### Differential diagnosis.

This species is very similar to *H.lentus* Sharp, 1890, *H.densus* Sharp, 1890, *H.sauteri* d’Orchymont, 1943 and *H.hainanensis* Dong & Bian, 2021 in size, form and other morphological characters. It can be distinguished based on aedeagus characters. Aedeagus: membranous inner sac with a cluster of strong sclerotized spines (Figs 24–26); median lobe with a lateroventral tooth subapically (Fig. 25).

#### Description.

**Figures 1–4.**
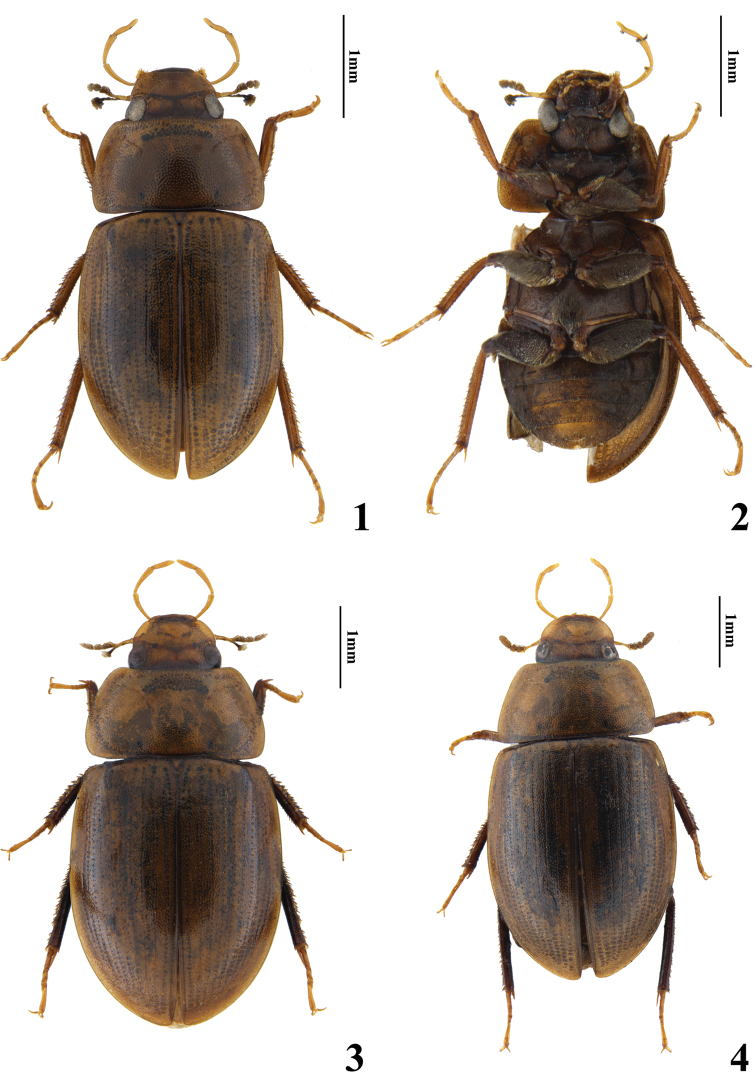
Habitus **1–2***Helocharesguoi* Yang & Jia, sp. nov. **1** dorsal view **2** ventral view **3–4***Helocharesdistinctus* Jia & Tang, sp. nov. **3** paratype, dorsal view **4** holotype, dorsal view.

***Form and colour*** (Figs 1–2, 6). Body length 4.0 mm, body width 2.1 mm. Oval, moderately convex. Dorsum of head, pronotum and elytron yellow-brown, clypeus black. Antennae yellow-brown with club black. Maxillary palps uniformly yellow-brown. Labial palps yellow, not darkened apically. Venter, including legs, blackish brown, tarsomeres yellow-brown.

***Head*.** Antenna with scape ca as long as antennomeres 2 and 3 combined (Fig. 8). Maxillary palps ca 1.25 × as long as width of head anterior to eyes; apical segment symmetrical, about same as penultimate in length (Fig. 9). Clypeo-labral margin curved medially. Labrum, frons and clypeus with systematic punctures (with setae) same size as ground punctures; frons and clypeus with ground punctation dense and coarse, distance between punctures 0.8–1.2× width of one puncture. Mentum subquadrate, with anterior margin strongly emarginate, slightly depressed medially, surface with some oblique wrinkles.

***Thorax*.** Ground punctation on pronotum and elytron similar to that on head, distance between punctures 0.8–1.2× width of one puncture; anterior margin with very fine bead. Elytron with short scutellary series of punctures and 10 striae, punctures in striae distinctly coarser than surrounding ground punctation; systematic punctures (with setae) same size as coarse punctures in striae. Prosternum moderately elevated medially, not tectiform or carinate medially, with a transverse groove anteriorly. Mesoventrite with small tubercle medially, not carinate medially. Metaventrite without glabrous area posteromedially. Femora densely pubescent, only glabrous at apex. Meso-, and metatarsomeres 1 to 4 with dense long setae ventrally, metatarsomeres with a fringe of long swimming-hairs dorsally. Protarsal claws in male somewhat stronger than in female and slightly angularly curved, bearing a blunt basal tooth; mesotarsal claws as protarsals, but only moderately curved with a blunt tooth; metatarsal claws only moderately curved, with a blunt basal tooth.

**Figures 5–7.**
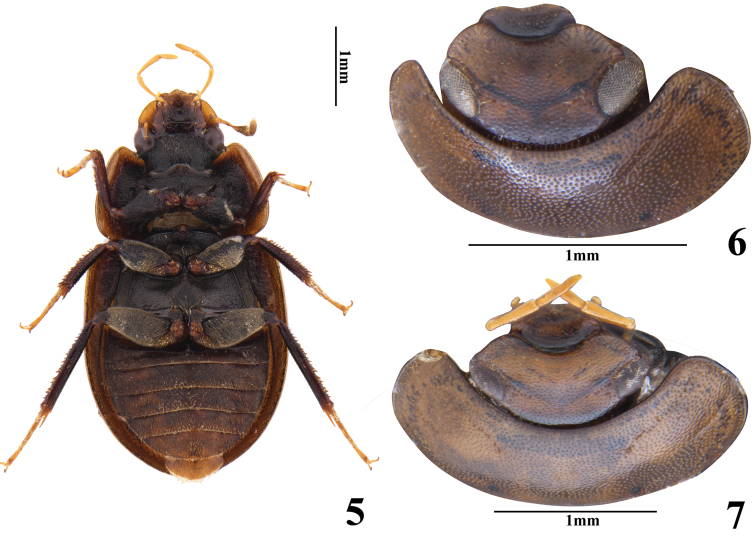
Habitus **5***Helocharesdistinctus* Jia & Tang, sp. nov.: ventral view **6–7** head, dorsal view **6***Helocharesguoi* Yang & Jia, sp. nov. **7***Helocharesdistinctus* Jia & Tang, sp. nov..

***Abdomen*.** Ventrites uniformly and densely pubescent. Fifth abdominal ventrite with apical emargination fringed with stiff yellowish setae.

***Aedeagus*** (Figs 24–26). Phallobase ca 0.12mm; paramere ca 0.76mm, obtuse apically, outer margin almost parallel in basal three quarters, apical quarter gradually narrowed and rounded apically; membranous inner sac with cluster of strong sclerotized spines (Figs 24–26); median lobe longer than parameres, ca 0.89 mm, apical fifth gradually narrowed apicad, with small latero-ventral tooth subapically, truncate apically (Fig. 25); basal apophyses about half as long as median lobe, ca 0.45 mm.

#### Remarks.

**Figures 8–20.**
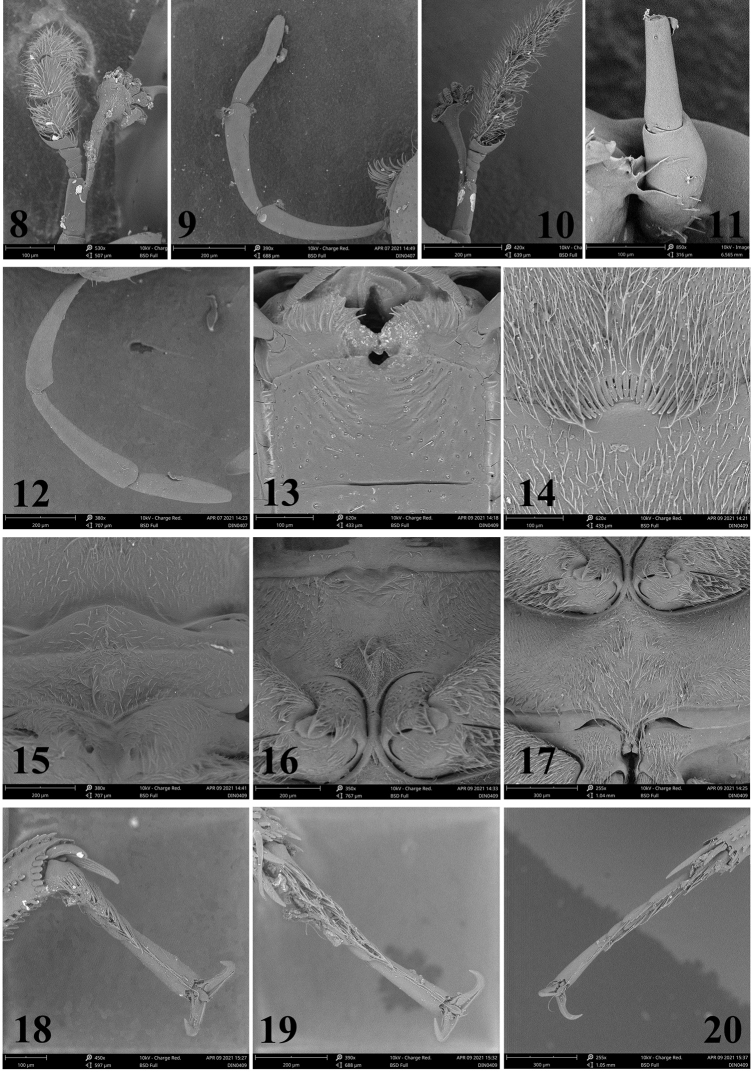
**8–9***Helocharesguoi* Yang & Jia, sp. nov. **8** antennae **9** maxillary palp **10–20***Helocharesdistinctus* Jia & Tang, sp. nov. **10** the antennae of fig3 **11** the antennae of fig4 **12** maxillary palp **13** mentum **14** apex of fifth abdominal ventrite **15** prosternum **16** mesoventrite **17** metaventrite **18** protarsomeres **19** mesotarsomeres **20** metatarsomeres.

The male holotype bears a long “branch” arising subapically from the antennal pedicel (Fig. 8), which is absent on the paratype (female). This structure is likely a fungus that parasitizes on the antenna.

#### Etymology.

This species is named after Mr. Qiang Guo, the manager of the Shenzhen Wildlife Conservation Division, Guangdong, for his help when we collected in Shenzhen.

#### Distribution.

China (Guangdong): known only from the type locality.

#### Habitat.

This species was collected in the mud at the edge of a seasonal pond.

### ﻿ Helochares distinctus

Taxon classificationAnimaliaColeopteraHydrophilidae

Jia & Tang sp. nov.

104C49D0-11C6-5BFD-96F8-0A501DC344CB

http://zoobank.org/385B9F5A-3203-4B21-902C-9FBC74DC07F6

Figs 3–4 , 5 , 7 , 10–20 , 21–23 , 27–28

#### Material examined.

***Holotype***: male, Jiangxi, Jing’an County, Zaodu town, Nanshan, 29°01'N, 115°16'E, 315m, 2.viii.2015, Renchao Lin and Yudan Tang leg. ***Paratype***: 1 male, Hunan, Guidong County, Bamianshan Nature Reserve, 25°58'21"N, 113°42'37"E, 973 m, 2015.vi.15, Renchao Lin and Yudan Tang leg.

#### Differential diagnosis.

This species is very similar to *H.lentus* Sharp, 1890, *H.densus* Sharp, 1890, *H.sauteri* d’Orchymont, 1943 and *H.hainanensis* Dong & Bian, 2021 in size, form and other morphological characters, but it is very easy to distinguish from all known species by aedeagal features. Aedeagus (Figs 27–28) with median lobe slightly shorter than parameres, nearly rhombic, apex with a globular structure with a cluster of apical spines and with a long baseball-bat-shaped branch medially, membranous inner sac with some strong spinous protrusions. *Helocharesdistinctus* Jia & Tang, sp. nov. can easily be distinguished from *H.guoi* Jia & Yang, sp. nov. by its larger size, median lobe of the aedeagus with a globular structure with a cluster of spines apically and with a long baseball-bat-shaped branch medially; membranous inner sac with some less strongly spinous protrusions.

#### Description.

***Form and colour*** (Figs 3–5, 7). Body length 5 mm, body width 2.6 mm, oval, moderately convex. Dorsum of head, pronotum, elytra and clypeus yellow-brown, labrum dark brown. Antennae yellow-brown with club black. Maxillary palps uniformly yellow-brown. Labial palps yellow, not darkened apically. Venter, including legs, blackish brown, tarsomeres yellow-brown.

***Head*.** Antennae with scape ca as long as antennomeres 2 and 3 combined (Figs 10–11). Maxillary palps ca 1.25 × as long as of width of head anterior to eyes; apical segment asymmetrical, slightly shorter than the penultimate in length (Fig. 12). Clypeo-labral margin straight medially. Labrum, frons and clypeus with systematic punctures (with setae) same size as ground punctures; frons and clypeus with ground punctation dense and coarse, distance between punctures 0.5–1.2× width of one puncture. Mentum subquadrate, with anterior margin strongly emarginate, slightly depressed medially, surface with some oblique wrinkles (Fig. 13).

**Figures 21–28.**
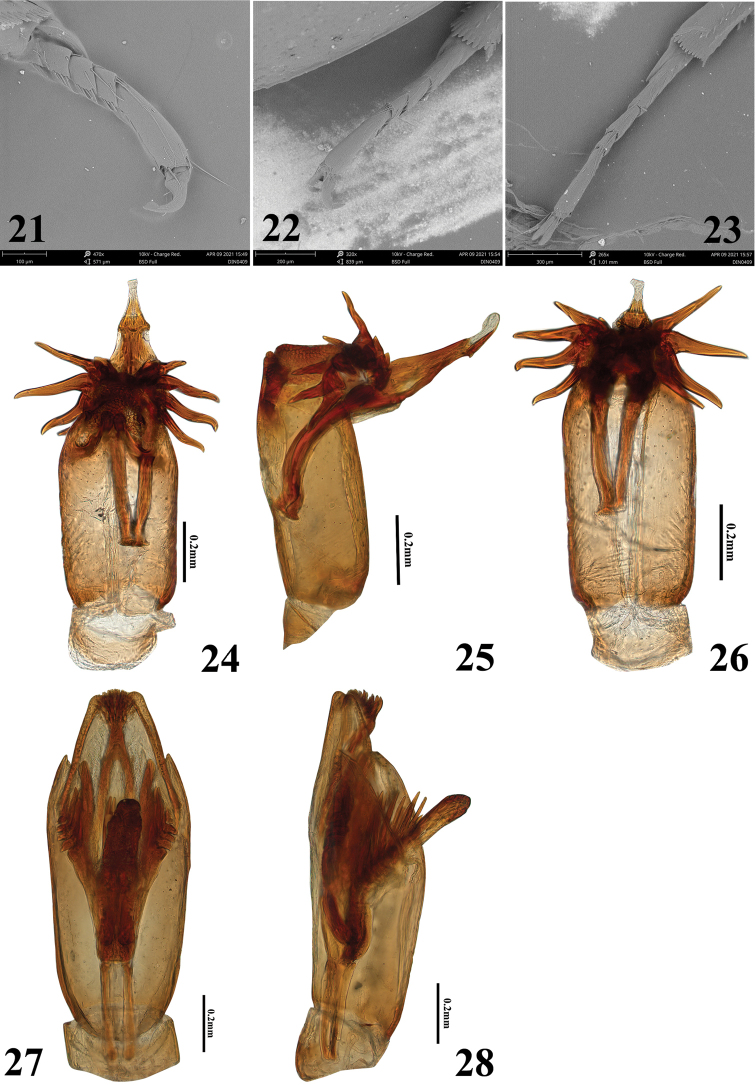
**21–23, 27–28***Helocharesdistinctus* Jia & Tang, sp. nov. **24–26***Helocharesguoi* Yang & Jia, sp. nov. **21** protarsomeres **22** mesotarsomeres **23** metatarsomeres **24–28** aedeagus **24** ventral view **25** lateral view **26** dorsal view **27** dorsal view **28** lateral view.

***Thorax*.** Ground punctation on pronotum and elytron similar to that on head, distance between punctures 0.5–1.2× width of one puncture; anterior margin without very fine bead. Elytron with short scutellary series of punctures and 10 striae, punctures in striae distinctly coarser than ground punctures; systematic punctures as coarse as punctures in striae. Prosternum moderately elevated medially, not tectiform or carinate medially (Fig. 15), with a transverse groove anteriorly. Mesoventrite with a small posteromedial tubercle, not carinate medially (Fig. 16). Metaventrite without glabrous area posteromedially. Femora densely pubescent except at apex (Fig. 17). Meso-, and metasomeres 1 to 4 with dense long setae ventrally, posterior tarsomeres with a fringe of long swimming-hairs dorsally. Protarsal claws in male somewhat stronger and a little angularly curved, bearing a blunt basal tooth; mesotarsal claws as protarsals, but only moderately curved with a blunt tooth; metatarsal claws only moderately curved, with a blunt basal tooth (Figs 18–23).

***Abdomen*.** Ventrites uniformly and densely pubescent. Fifth (apical) abdominal ventrite with apical emargination fringed with stiff yellowish setae (Fig. 14).

***Aedeagus*** (Figs 27–28). Phallobase ca 0.24 mm; paramere ca 1.0 mm, widest at the apical quarter, apical quarter slightly narrowed towards to the apex, apex rounded; median lobe slightly shorter than the parameres, ca 0.91 mm, nearly rhombic, apex with a globular structure with a cluster of apical spines and with a long baseball-bat-shaped branch medially; membranous inner sac with some strong spinous protrusions; basal apophyses about one third as long as the median lobe, ca 0.35 mm.

#### Remark.

The antennal pedicel of the male paratype also bears a long fungus as in *H.guoi* Yang & Jia sp. nov. (Fig. 10). However, the male holotype of this species lacks such a structure, although only the scape and pedicel remained on the right antenna (Fig. 11) and the left antenna was lost.

#### Etymology.

Latin “distinctus”, referring to the antennae and aedeagus with clearly different characters from other known species.

#### Distribution.

China (Jiangxi, Hunan).

#### Habitat.

Living on edge of stagnant water pool.

#### Additional faunistic data.

Fig. 65

### ﻿ Helochares hainanensis

Taxon classificationAnimaliaColeopteraHydrophilidae

Dong & Bian, 2021

FC13FCB7-7875-5F37-9591-50F0E4D33673

**Figures 29–32.**
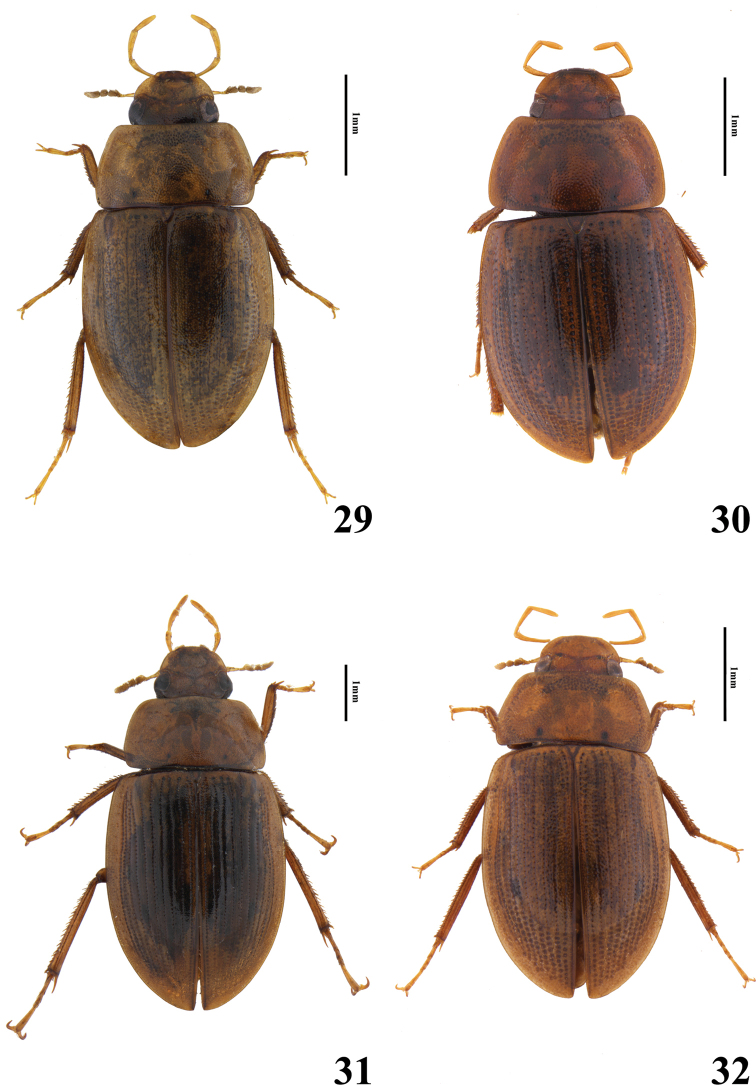
Habitus of *Helochares* spp., dorsal view **29***H.hainanensis* Dong & Bian **30***H.nipponicus* Hebauer **31***H.negatus* Hebauer **32***H.minusculus* d’Orchymont.

Figs 29 , 38 , 45 , 46

Helochares (Hydrobaticus) hainanensis Dong & Bian, 2021:168. Type locality: China (Hainan).

#### Material examined.

**Guangdong**: 6 males, 11 females, Shenzhen, Dapeng Peninsula, Kuichong, Paiyashan Mt., alt. 8 m, 22°38'59"N, 114°30'37"E, 5.xi.2018, Weicai Xie leg.

#### Distribution.

China (Hainan, Guangdong). New for Guangdong.

#### Habitat.

This species occurs in mud with aquatic grass at the edge of a pool.

### ﻿ Helochares nipponicus

Taxon classificationAnimaliaColeopteraHydrophilidae

Hebauer, 1995

5CA6760E-8546-5B0C-BE7D-9308A2DD8284

Figs 30 , 47 , 48

Helochares striatus Sharp, 1873: 60. Type locality: Japan (Kyushu).
Helochares nipponicus Hebauer, 1995: 6 (RN). Species name “striatus” was preoccupied by Hydrobiusstriatus Boheman, 1851 (= Helocharesstriatus (Boheman 1851)).

#### Material examined.

**Nei Mongol**: 1 male, Tongliao, The source of Daqinggou, 235 m, 27.viii.2014, Weijie Sun leg. **Jiangxi**: 1 male, Shangrao, Sanqingshan, 15–20.iv.2007, Fenglong Jia leg.

#### Distribution.

China (Jilin, Nei Mengol, Jiangxi), Japan, Korea. New for Jiangxi and Nei Mongol.

#### Habitat.

This species occurs in mud with aquatic grass at the edge of a pool.

### ﻿ Helochares negatus

Taxon classificationAnimaliaColeopteraHydrophilidae

Hebauer, 1995

8FF0EC9E-DF59-5088-8ED1-E6CE39259AC8

Figs 31 , 39 , 49 , 50

Helochares negatus Hebauer, 1995b: 5. Type locality: Bangladesh (Dinajpur).

#### Material examined.

**Yunnan**: 2 males, 3 spec., Mengla, 4.viii.2007, Jiahui Li leg., 1 male, Mengla, Wangtianshu, 22.vii.2011, Yun Li leg., 1 male., Puer, 29.vii.2007, Fenglong Jia leg.; 5 males, 2 females, Yingjiang County, Tongbiguan village, Kaibangyahu, 24.58°N, 97.67°E, 1289 m, 25.v.2016, Yudan Tang and Ruijuan Zhang leg.

#### Distribution.

China (Yunnan), Bangladesh. New for China.

#### Habitat.

This species occurs in mud with aquatic grass at the edge of a pool. It is occasionally collected by light trap.

### ﻿ Helochares minusculus

Taxon classificationAnimaliaColeopteraHydrophilidae

d’Orchymont, 1943

3C240B98-D305-500D-A071-562F67E9ED79

Figs 32 , 40 , 51 , 52

Helochares minusculus d’Orchymont, 1943a: 10. Type locality: Indonesia (Sumatra).

#### Material examined.

**Guangdong**: 3 males, 44 spec., Zhuhai, 24.xi.2007, Fenglong Jia leg.; 4 spec., Zhuhai, Qi’ao Island, 12.VII.2005, Fenglong Jia leg.; 1 male, Shaoguan, Danxiashan, 27.v.2010, Fenglong Jia leg.

#### Distribution.

China (Guangdong), Myanmar, Indonesia. New for China.

#### Habitat.

This species occurs in mud with aquatic grass at the edge of pool or slow stream.

### ﻿ Helochares sauteri

Taxon classificationAnimaliaColeopteraHydrophilidae

d’Orchymont, 1943

27607449-D8E2-582B-ACBC-395D4B9F14D4

**Figures 33–36.**
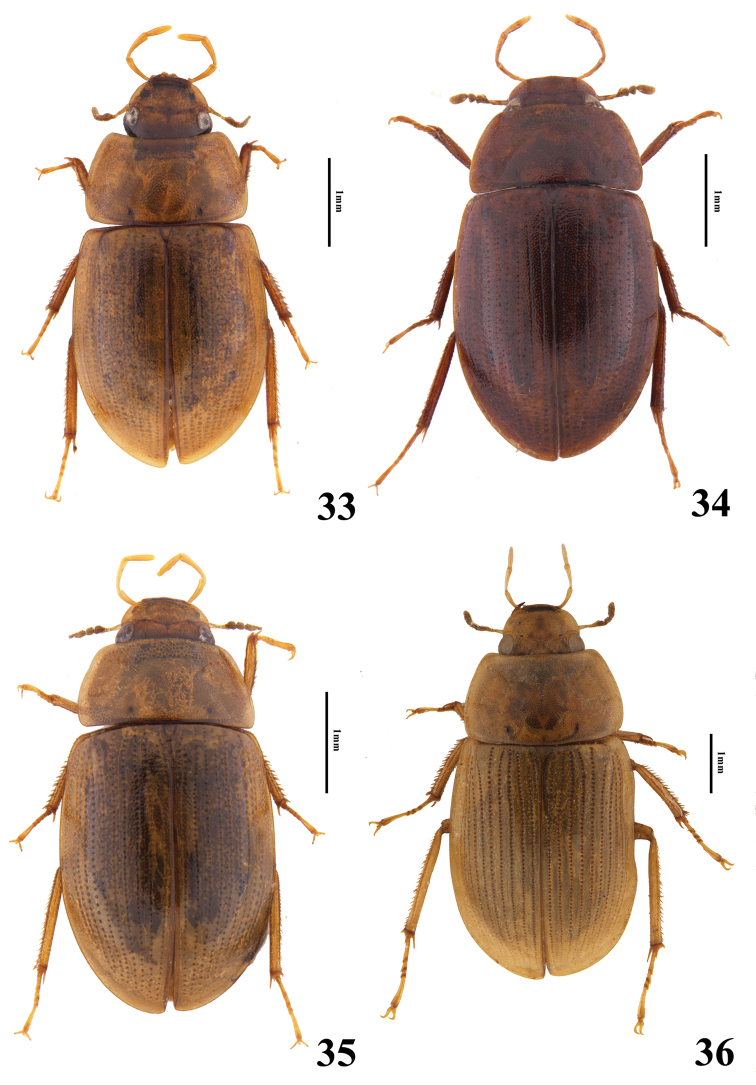
Habitus of *Helochares* spp., dorsal view **33***H.sauteri* d’Orchymont **34***H.densus* Sharp **35***H.lentus* Sharp **36***H.neglectus* (Hope).

Figs 33 , 41 , 53 , 54 , 55

Helochares Sauteri d’Orchymont, 1943a: 6. Type locality: China (Taiwan).

#### Material examined.

***Paratype*** male (IRSN), Ta-maon Id. (II), 92–87. A. d’Orchymont det.: Helochares (Hydrobaticus) sauteri m., coll. A. d’Orchymont.

**Figures 37–40.**
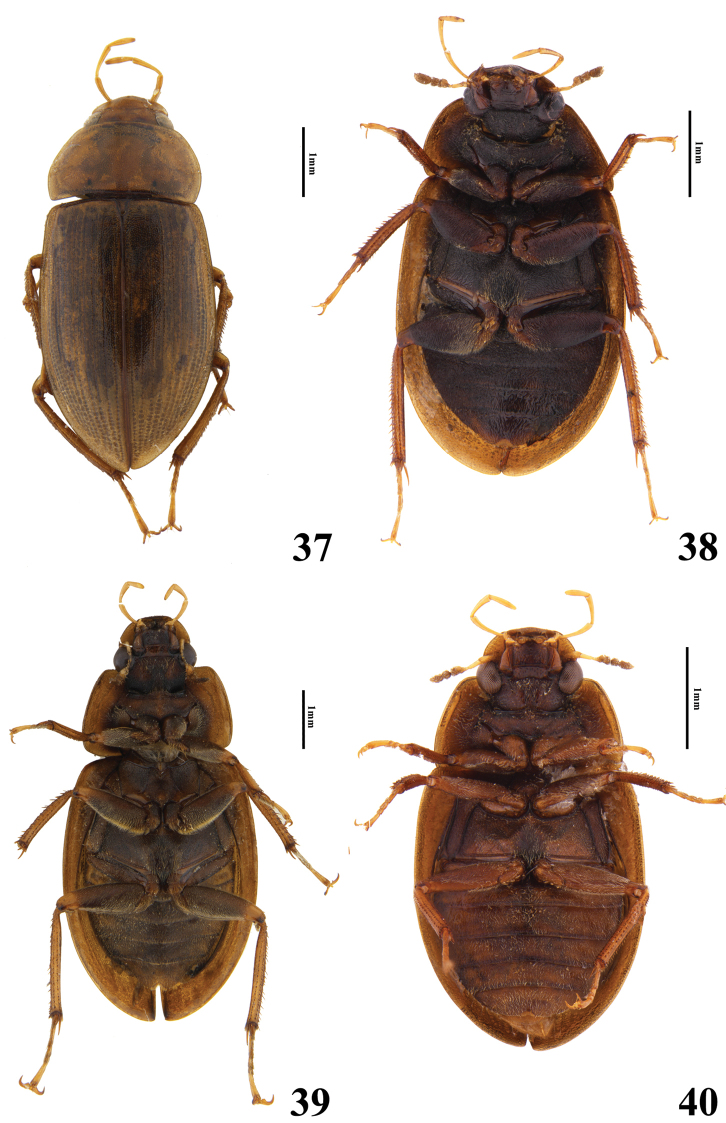
Habitus of *Helochares* spp. **37***H.anchoralis* Sharp (dorsal) **38***H.hainanensis* Dong & Bian (ventral) **39***H.negatus* Hebauer (ventral) **40***H.minusculus* d'Orchymont, (ventral).

**Figures 41–44.**
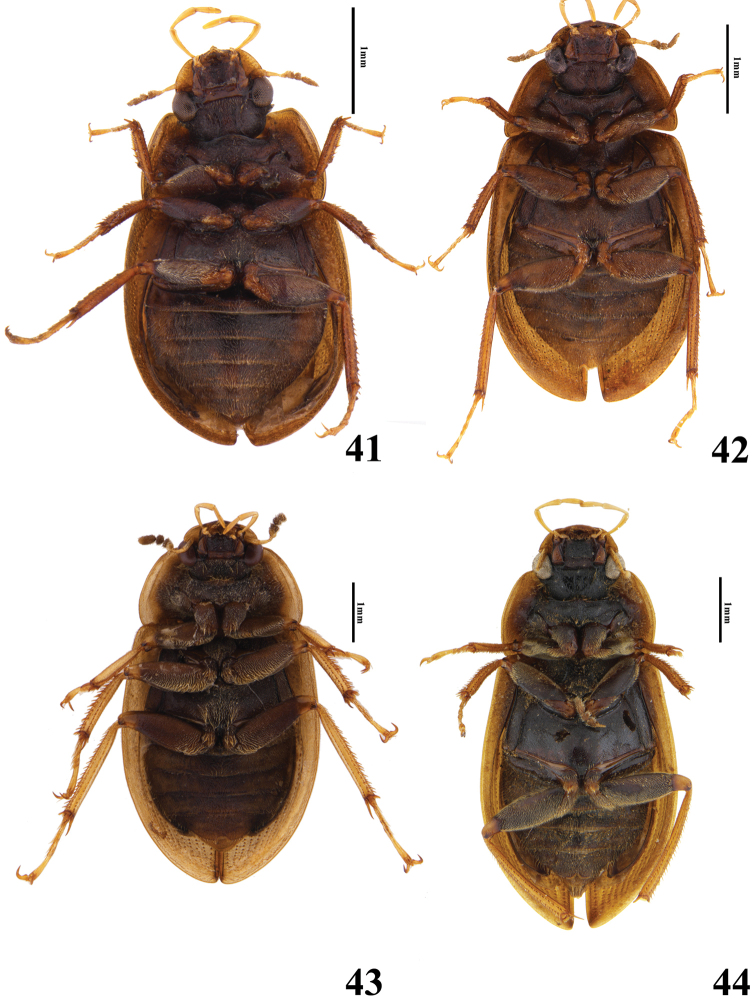
Habitus of *Helochares* spp **41***H.sauteri* d’Orchymont (ventral) **42***H.lentus* Sharp (ventral) **43***H.neglectus* (Hope) (ventral) **44***H.anchoralis* Sharp (ventral).

**Figures 45–52.**
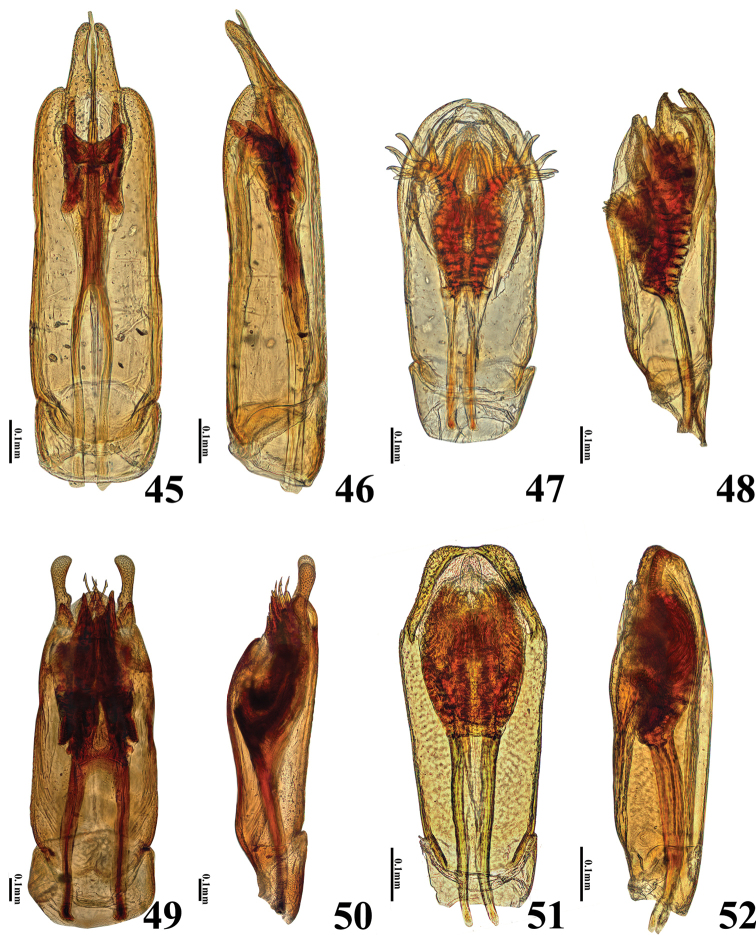
Aedeagi of *Helochares* spp **45–46***H.hainanensis* Dong & Bian **45** dorsal **46** lateral **47–48***H.nipponicus* Hebauer **47** dorsal **48** lateral **49–50***H.negatus* Hebauer **49** dorsal **50** lateral **51–52***H.minusculus* d’Orchymont **51** dorsal **52** lateral.

#### Additional material examined.

**Hubei**: 1 spec., Wuchang, 17.v.1961, Zhelong Pu leg. **Zhejiang**: 55 spec., Tianmushan, 27.vii.-10.viii.2009, Fenglong Jia leg. **Jiangxi**: 1 male, 5 females, Yichuan City, Yifeng County, Guanshan nature reserve, 26°30'05.63"N, 114°00'53.19"E, 379 m, 17–18.vi.2016, Yudan Tang and Ruijuan Zhang leg.; 11 spec., Jiulianshan, 20.iv.2009, Fenglong Jia leg.; 42 spec., Shangrao, Sanqingshan, 15.viii.2006 & 15–20.iv.2007, Fenglong Jia and Haidong Chen leg.; 4 spec., Jinggangshan, Baiyinghu, 800m, 27.iv.2011, Fenglong Jia leg.; 6 spec., Jinggangshan, Shuangxikou, 3.x.2010, Shuang Zhao and Fenglong Jia leg.; 2 spec., Jinggangshan, Dajing parkland, 19.ix.2010, Shuang Zhao leg.; 3 spec., Jinggangshan major peak, 2.x.2010, Yue Jia and Yuran Cao leg.; 1 spec., Jinggangshan, Jingzhushan, 4.x.2010, Fenglong Jia leg.; 21 spec., Jing’an County, Sanzhaolun village, Tangli, 260 m, 3.viii.2015, Renchao Lin and Yudan Tang leg.; 22 spec., Suichuan County, Nanfengmian nature reserve, 816 m, 18.vi.2015, Renchao Lin and Yudan Tang leg.; 17 spec., Jing’an County, Daqishan forestry centre, 350 m, 16.vii.2014, Renchao Lin leg.; 6 spec., Jing’an County, Zaodu town, Nanshan village, 315 m, 19.vii.2014, Renchao Lin leg.; 1 male, 5 spec., Shangyou County, Guanggushan, 25°55'11"N, 114°03'04"E, 846 m, 21.vi.2015, Renchao Lin and Yudan Tang leg. **Hunan**: 1 male, 3 females, Hunan, Zhuzhou City, Taoyuandong nature reserve, 28°33'16.73"N, 113°34'55.97"E, 394 m, 14–15.vi.2016, Yudan Tang and Ruijuan Zhang leg.; 2 spec., Nanyue, 4.ix.1941, Zhelong Pu leg.; 3 spec., Zhuzhou City, Yanling County, Taoyuandong, Jiashui, 19.v.2014, Renchao Lin and Xiaolin Liu leg.; 2 spec., Zhuzhou City, Yanling County, Taoyuandong, Mihua village, 25.v.2014, Renchao Lin and Xiaolin Liu and Chang Pan leg.; 1 spec., Zhuzhou City, Yanling County, Taoyuandong, 20.v.2014, Xiaolin Liu and Chang Pan and Weicai Xie leg. **Fujian**: 4 spec., Wuyishan, Daanyuanhe, 16.vii.2010, Fenglong Jia leg.; 14 spec., Nanjing, Hexi town pond, 13.vii.2010, Fenglong Jia leg.; 1 spec., Ningde City, Ningde normal college behind the mountain, 200 m, 3.x.2012, Zeyu Wang leg. **Guangdong**: 40 spec., Shaoguan, Danxiashan, 20.iv.2008 & 16.V.2009 & 27.V.2010 & 28.VIII.2012 & 23–26.IV.2013, Fenglong Jia and Keqing Song and Shuang Zhao leg.; 3 spec., Danxiashan, Zhanglaofeng, 8.vi.2012, Fenglong Jia leg.; 2 spec., Danxiashan, Yangyuanshan, 10.vi.2011, Fenglong Jia leg.; 1 spec., Danxiashan, Jinshiyan, 22.iv.2012, Fenglong Jia and Junlei Liao leg.; 2 spec., Fengkai, Heishiding, 20–22.vii.2007, Fenglong Jia and Lijun Yang leg.; 2 spec., Fengkai, Heishiding, 2.vii.2011, Fenglong Jia and Lijun Yang leg.; 4 spec., Nanling, Dadongshan, 24.vi.2009, Fenglong Jia leg.; 2 females, Guangzhou, Baiyunshan, 18.iv.1958, Zhelong Pu leg.; 8 spec., Huizhou, Longmen County, Nankunshan, 23.6538N 113.9469E, 239.6 m, 26.ix.2021, Zhuoyin Jiang and Zuqi Mai leg. **Guizhou**: 2 spec., Pingba, Machang, 13.viii.1982, Zhihe Huang leg.; 2 spec., Rong County, Pingyang village, Xiaodanjiang, 15.ix.2005, Shuang Zhao leg. **Sichuan**: 6 spec., Leshan City, Emeishan, Qingyin’ge, 750 m, 7.vi.2014, Renchao Lin leg.; 2 spec., Emeishan, 6.vii.1982, Zhihe Huang leg.; 3 spec., Qingchengshan, 8.viii.1982, Zhihe Huang leg.

#### Distribution.

China (Fujian, Guangdong, Guizhou, Hubei, Hunan, Jiangxi, Sichuan, Taiwan, Zhejiang). New for Hunan.

#### Habitat.

This species occurs in mud, or under root of waterside grass of pool or slow stream. It can occasionally be collected in mud without aquatic grass, and by light trap.

**Figures 53–60.**
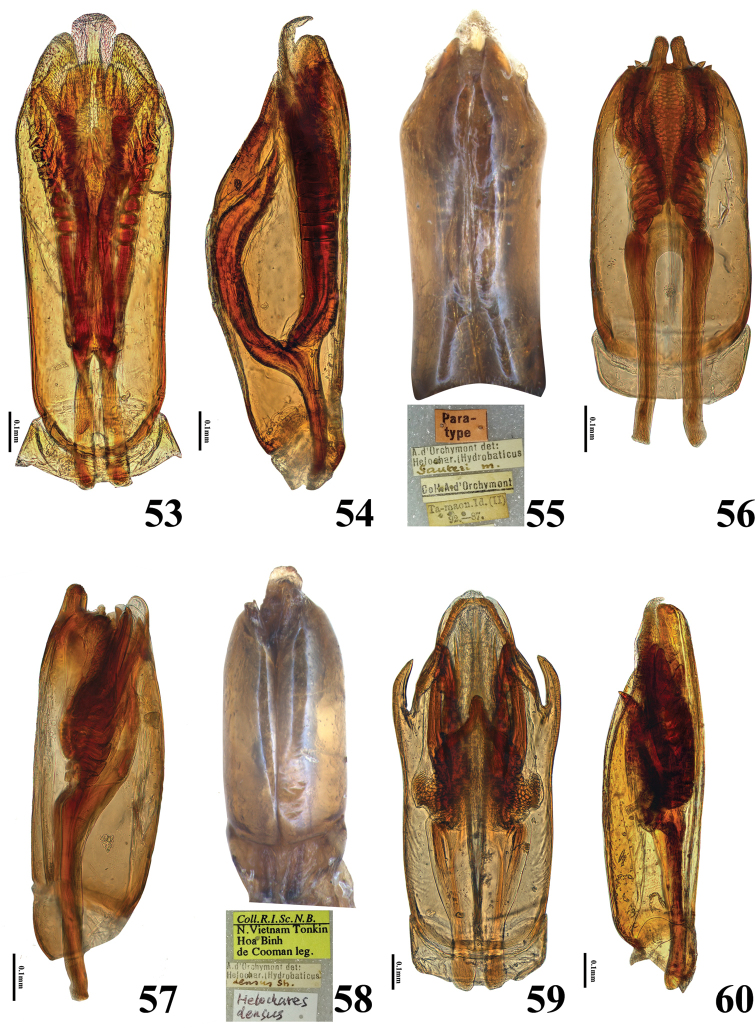
Aedeagi of *Helochares* spp **53–55***H.sauteri* d’Orchymont **53** dorsal, **54** lateral, **55** ventral and data of specimen **56–58***H.densus* Sharp **56** dorsal **57** lateral **58** ventral and data of specimen **59–60***H.lentus* Sharp **59** dorsal **60** lateral.

### ﻿ Helochares densus

Taxon classificationAnimaliaColeopteraHydrophilidae

Sharp, 1890

9098D108-AEA8-5287-B531-E61F011AF17B

Figs 34 , 56 , 57 , 58

Helochares densus Sharp, 1890: 352. Type locality: Sri Lanka (Kandy; Dikoya; Bogawantalawa).

#### Material examined.

1 male (IRSN), coll. R.I.Sc.N.B. N. Vietnam Tonkin, Hoa Bih, de Cooman leg.; A. d’Orchymont det.: *Helochar*. (*Hydrobaticus*) *densus* sb., with a handwriting label: *Helocharesdensus*.

#### Additional material examined.

**Guangdong**: 4 males, 3 females, Shenzhen, Dapeng Peninsula, Kuaichong, Paiyashan Natural Park, 22°38'59"N, 114°30'37"E, alt. 8 m, 5.xi.2018, Fenglong Jia and Weicai Xie leg.; 1 male, Shenzhen, Dapeng Peninsula, Bantianyun, 22°31'16"N, 114°29'43"E, alt. 127.73 m, 7.viii.2019, Zhenming Yang, Zhuoyin Jiang, Guangyu Guo and Xinyuan Ji leg.; 4 spec., Shenzhen, Paiyashan, 17.v.2012, Fenglong Jia and Junlei Liao leg.; 3 spec., Shenzhen, 8–15.viii.2006, Fenglong Jia leg.; 1 male, 2 females, Shenzhen, Pingshan, Malanshan, 22°38'31"N, 114°19'41"E, alt. 284 m, 27.vii.2019, Zhenming Yang, Zhuoyin Jiang, Guangyu Guo and Xinyuan Ji leg. Shenzhen, Neilingding, 10.v.1998, Tongxu Peng leg.; 9 spec.; 9 spec., Zhuhai, 24.xi.2007, Fenglong Jia leg.; 3 spec., Zhuhai, the mountain behind of campus of Sun Yat-sen University, 5–8.vii.2011, Fenglong Jia leg.; 5 spec., Zhuhai, Hengqin Island, 10.vii.2006, Fenglong Jia leg.; 4 spec., Zhuhai, Qi’ao Island, 12.vii.2005, Fenglong Jia leg.; 2 spec., Danxiashan, Jinshiyan, 8.vi.2012, Fenglong Jia leg.; 1 spec., Danxiashan, the north of Yangyuanshi paddyfield, 23.iv.2012, Junlei Liao leg.; 1 spec., Xinhui, 6.iv.2006, Fenglong Jia leg.; 20 spec., Guangzhou, Baiyunshan, 23.1978N 113.2948E, 15.ix.2021, Zhuoyin Jiang and Zuqi Mai leg.; 1 male, 2 females, Guangzhou, Kangle, 24.vii.1964, Jiuru Zhang leg.; 1 spec., 1 spec., Shantou, 15.v.1964, Tongxu Peng leg. **Guangxi**: 1 female, Jingxi, Bangliang, 6.viii.2010, Jianhua Huang leg. **Hainan**: 4 spec., Jianfengling, 22.xi.1983, Zhihe Huang leg.; 2 spec., Jianfengling, Tianchi, 5–6.vii.1981, Guofeng He leg.; 3 spec., Wanning, 17.xii.1957, Cuiying Li leg.; 2 spec., Tongshi, 19.xii.1957, Cuiying Li leg.; 1 spec., Xinglong, 3.i.1964, Tongxu Peng leg.; 6 spec., Changjiang, Bawang town, 10.v.2007, Yibing Ba and Juntong Lang leg. **Macao**: 1 male, Ludangcheng, ecological preservation area, one area, 15–16.x.2016, Fenglong Jia and Weicai Xie leg. **Yunnan**: 2 spec., 1090 m, 30.vii.2010, Wangang Liu leg.; 1 female, Honghehekou, Binglangzhai Reservoir, 4.v.2011, Yun Li light trap.; 1 spec., Mengla, Wangtainshu, 6–7.viii.2007, Guodong Ren and Wenjun Hou and Yalin Li leg.; 1 male, Xishuangbanna Botanical Garden (west area), near Wanglian Hotel, 4–11.iv.2021, Huang Baoping leg.

#### Distribution.

China (Fujian, Guangdong, Guangxi, Hainan, Hunan, Jiangxi, Macau, Sichuan, Yunnan, Zhejiang), Andaman Islands, India, Thailand, Vietnam. New for Macao.

#### Habitat.

This species occurs in mud with aquatic grass at the edge of pool, under root of waterside grass, or slow stream. It never was collected by light trap.

### ﻿ Helochares lentus

Taxon classificationAnimaliaColeopteraHydrophilidae

Sharp, 1890

644A4248-2EB1-555D-B5A3-58C5B83DF603

Figs 35 , 42 , 59 , 60

Helochares lentus Sharp, 1890: 352. Type locality: Sri Lanka (Dikoya).

#### Material examined.

**Guangdong**: 1 male, 1 spec., Xuwen, 27.ix.1985, Zhihe Huang leg.; 1 spec., Zhanjiang, Chikan, 25.ix.1985, Zhihe Huang leg.; 10 spec., Fengkai, Heishiding, 13.viii.2010, Fenglong Jia, Yue Jia, Bingjie Chen and Weilin Xu leg.; 12 spec., Fengkai, Heishiding, 4–6.x.2013, Fenglong Jia, Yue Jia, Bingjie Chen and Weilin Xu leg.; 8 spec., Fengkai, Heishiding, 20–22.ix.2014, Fenglong Jia, Renchao Lin and Yudan Tang leg.; 2 spec., Fengkai, Heishiding, 29.v.1984, Wu Wu leg.; 2 spec., Fengkai, Heishiding, 10.iv.1985, Zhihe Huang leg.; 2 males, Guangzhou, Conghua, Liuxihe, Xitou village, 23.7125N 113.8697E, 398.6 m, 28.ix.2021, Zhuoyin Jiang and Zuqi Mai leg.; 4 spec., Shenzhen, Futian mangrove salt-water fish pond, 30.v.2015–1.vi.2015, Fenglong Jia and Renchao Lin leg. **Guangxi**: 6 spec., Fangcheng, Fulong, 24.v.1999, Xin Ke leg.; 3 spec., Napo, Nonghua, 750 m, 18.viii.1998, Fusheng Huang leg.; 1 spec., Jinxiu, Luoxiang, 200 m, 15.V.1999, Xuezhong Zhang leg. **Hong Kong**: 21 spec., Qingkuai pond, 29.x.2013. Y.M. Lee and Eric and Rex Ch Shih and Alex Lee leg.; 32 spec., Rongshuao, 10 m, 11.vi.2014, Fenglong Jia and Weicai Xie and Jiahuang Chen leg.; 2 spec., Nanyong (before the dam), 21 m, Fenglong Jia and Weicai Xie and Alex leg.; 2 spec., Shaluodong, 185 m, 28.x.2013, Fenglong Jia and Weicai Xie and Alex light trap. **Jiangxi**: 1 spec., Jing’an County, Zaodu town, Nanshan village, 315 m, 2.viii.2015, Renchao Lin and Yudan Tang leg. **Xizang**: 1 male (IZCAS), IOZ(E)2056679, Motuo County, Beibeng, near Liberation Bridge, 2016.VI.17N [N = night], 773 m, 29.2432°N, 95.1673°E, Liang Hongbin leg.; 1 male, 3 spec. (IZCAS), Motuo County, Beibeng, 2015.VIII.23N [N = night], 799 m, light trap, 29.3431°N, 95.1700°E, Liang Hongbin and Huang Zhengzhong leg. **Yunnan**: 6 spec., Yingjiang County, Nabang town, 24.75°N, 97.56°E, 239 m, Yudan Tang and Ruijuan Zhang leg.; 50 spec., Jingdong County, Taizhong town, 1395 m, 15.iv.2015, Renchao Lin and Yudan Tang leg. ; 2 spec., Pohui, 2.ix.1939, Zhelong Pu leg.; 2 spec., Lufeng village, 26.iii.1940.; 1 female, Jinping, Mengla, 370 m, 30.iv.1956, Keren Huang leg.; 1 female, Jingdong, 1170 m, 24.vi.1956, Keleirangnuofusiji leg.; 1 female, Hekou, Xiaonanxi, 200m, 7.vi.1956, Keren Huang leg.; 1 female, Cheli, Damenglong, 640 m, 29.iv.1957, Shuyong Wang leg.; 1 female, Mangshi, 1000 m, 12.v.1956, Benshou Zhou leg.; 4 spec., Mengla, 2007.viii.2, Jiahui LI leg.; 2 spec., Mengla Shangyong, 2007.viii.2, Lei Shi leg.; 1 spec., Mengla, 6–7.viii.2007, Guodong Ren, Wenjun Hou and Yaping Li leg.; 1 spec., Wangting, 2011.iv.29, Wangang Liu leg.; 2 spec., Yingjiang, 820 m, 25.v.1983, Lizhong Hua leg.; 2 spec., Huijiang, i.1940, Zhelong Pu leg. 3 males, 1 female, Xishuangbanna Botanical Garden (west area), near Wanglian Hotel, 4–11.iv.2021, Huang Baoping leg.; 1 male, 1 female, Honghe, Hani Automatic prefecture of Yi Nationality, Lvchun County, Niukong town, in Terrance, 1336 m, 22.9872°N, 102.2675°E, Jiang Zuoyin, Yang Zhenming, Mai Zuqi and Huang Baoping leg.

#### Distribution.

China (Fujian, Guangdong, Guangxi, Guizhou, Hong Kong, Hunan, Jiangxi, Sichuan, Taiwan, Xizang, Yunan), Bangladesh, Cambodia, India, Indonesia, Malaysia, Sri Lanka, Thailand, Vietnam. New for Fujian, Guangdong, Guizhou, Hunan, Jiangxi, Sichuan.

#### Habitat.

This species occurs in mud with aquatic grass at the edge of a pool. It can occasionally be collected by light trap.

**Figures 61–64.**
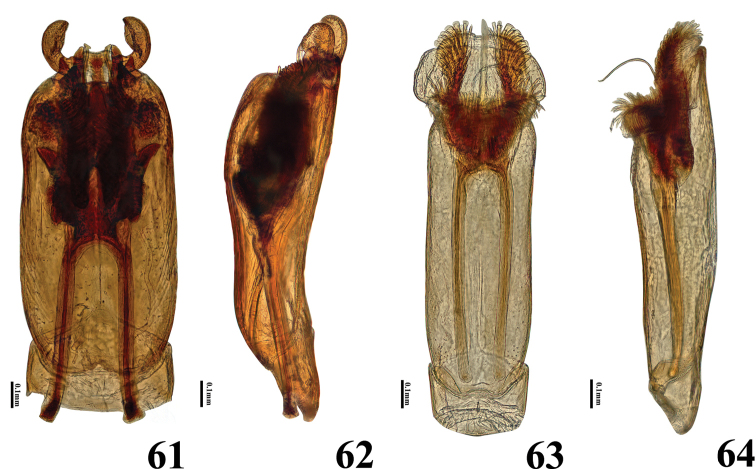
Aedeagi of *Helochares* spp **61–62***H.neglectus* (Hope) **61** dorsal **62** lateral **63–64***H.anchoralis* Sharp **63** dorsal **64** lateral.

### ﻿ Helochares neglectus

Taxon classificationAnimaliaColeopteraHydrophilidae

(Hope, 1854)

85DE4841-A718-5206-9535-F53CA25412FC

Figs 36 , 43 , 61 , 62

Hydrobius neglectus Hope, 1854: 16. Type locality: Guangzhou, China.
Helochares crenatus (Régimbart, 1903): Pu 1963: 79 (misidentification, Yunnan).

#### Material examined.

**Jiangxi**: 30 spec., Jing’an County, Zaodu town, Nanshan village, 315 m, 29°01'N, 115°16'E, 2.viii.2015, Renchao Lin and Yudan Tang leg.; 5 spec., Jing’an County, Zaodu town, Nanshan village, 315 m, 29°01'N, 115°16'E, 2.viii.2015, Renchao Lin and Yudan Tang leg.; 32 spec., Jing’an County, Zaodu town, Nanshan village, 315 m, 29°01'N, 115°16'E, 19.vii.2015, Renchao Lin and Yudan Tang leg.; 1 spec., Lushan, Poyanghu, 10.viii.1963, Zhelong Pu leg.; 1 spec., Jiujiang, 24.viii.1941, Zhelong Pu leg. **Hunan**: 1male, 8 spec., Jishou City, Mayang County, Lancui village, 27°46'17"N, 109°51'41"E, 349 m, 15.ix.2016, Fenglong Jia and Ruijuan Zhang leg.; 3 spec., Yizhang, 8.x.1941, Zhelong Pu leg.; 2 spec., Tongdao, 19.viii.1982, Zhihe Huang leg.; 2 spec., Xianghuaihua, Yushuwan, 17.vi.1965, Zhenyao Chen leg.; 1 spec., Nanyue, 4.ix.1941, Zhelong Pu leg.; 1 spec., Chengyuan, 6.iii.1941, Zhelong Pu leg. **Fujian**: 1 spec., Fu’an, 20.ix.1963, Shanxiang Lin leg. **Guangdong**: 1 sepc., Shenzhen, Neilingding nature reserve, 22°24'44"N, 113°48'46"E, 6m, 23–26.viii.2016, Fenglong Jia, Weicai Xie, Ruijuan Zhang and Shishuai Wang leg.; 27 spec., Baiyunshan, 2.xi.1964, Jincai Bao leg.; 20 spec., Guangzhou, Luhu, 2.xi.1964, Zhenyao Chen leg.; 32 spec., Lianhe, 18.x.1964, Zhenyao Chen and Jincai Bao leg.; 20 spec., Guangzhou, Xinshi, 11.x.1964, Chengmu Chen and Zhengwei Huang leg.; 10 spec., Heshan, 22–24.iv.2002, Ruizhen Wen leg.; 7 spec., Heshan, 6.vi.2006, Guilin Liu leg.; 8 spec., Dongguan, Lianhuashan, 20.vi.2002, Guilin Liu leg.; 12 spec., Lianzhou, Dadongshan, 25.ix.2008, Yun Wang leg.; 2 spec., Dinghu, 10.v.1994, Fenglong Jia leg.; 1 spec., Dinghushan, 22–23.v.1964, Ping Lin and Yaoquan Li leg.; 1 spec., Xuwen, 27.ix.1985, Zhihe Huang leg.; 1 spec., Henan, Kangle, 30.vi.1964 & 13.vii.1964, Qiuquan Li, Jiuru Zhang, Shitian Li and Shunbang Liu leg.; 6 spec., Henan, Kangle, 2.vii.1965, Qiuquan Li, Jiuru Zhang, Shitian Li and Shunbang Liu leg.; 2 spec., Henan, Kangle, xii.1962, Qiuquan Li, Jiuru Zhang, Shitian Li and Shunbang Liu leg.; 1 spec., Xinhui, viii.2001, Xiaoli Tong leg.; 4 spec., Guangzhou, viii.1938, Zhelong Pu leg.; 6 spec., Guangzhou, Henan, 24.v.1957, Zhelong Pu leg.; 1 spec., Guangzhou, Xicun, 3.iv.1963, Yousheng Lai leg.; 1 spec., Sun Yat-sen University campus, 26.iv.1963, Youzheng Lai leg.; 2 spec., Sun Yat-sen University campus, vii.1985, Youzheng Lai leg.; 2 spec., Guangzhou, Shipai, 26.vi.1955, Zhaojian Liang leg.; 2 spec., Guangzhou, Chisha, 28.ix.1964, Zhenyao Chen leg.; 3 spec., Guangzhou, Shipai, 18.x.1964, Zhaojian Liang leg.; 1 spec., Guangzhou, Ruyuan, Longxi, 9.x.1964, Zhenyao Chen leg.; 1 female, Guangzhou, Shuzhugang, 3.v.1957, Zhelong Pu leg.; 1 female, Shenzhen, 12–15.viii.2006, Fenglong Jia leg.; 10 spec., Shenzhen, Futian Mangrove nature reserve, 2–4.iv.2015, Fenglong Jia, Renchao Lin, Zhenhua Liu and Kai Chen leg.; 7 spec., Shenzhen, Futian Mangrove nature reserve, 30.v-1.vi.2015, Fenglong Jia, Renchao Lin, Yudan Tang and Kai Chen leg.; 1 spec., Shenzhen, Luohu, Yinhu, 28.xi.1998, Fenglong Jia leg.; 2 spec., Lianxian, vi.1945, Zhelong Pu leg.; 1 spec., Fengkai, Heishiding, 1.vii.1987, Chen leg.; 1 female, Gaoming, Yangmei town, 23–26.iv.2006, Fenglong Jia leg.; 1 spec., Yingde, 5.viii.1962, Ping Lin leg.; 1 spec., Guangzhou, Xinzhou, 17.vi.1963, Youshen Lai leg.; 1 spec., Guangzhou, Shilangang, 9.v.1963, Youshen Lai leg. **Guangxi**: 98 spec., Yangshuo, 1985, Shoujian Chen leg.; 3 spec., Nanning, 19.vi.1977, Zhelong Pu leg.; 10 spec., Nanning, vi.1958, Zhihe Huang leg.; 11 spec., Shangsi, Hongqi forestry centre, 300 m, 27.v.1999, Xuezhong Zhang leg.; 5 spec., Fangcheng, Fulong, 23.v.1999. Xin Ke leg.; 3 spec., Napo, Nonghua, 750m, 18.viii.1998, Fusheng Huang and Wenzhu Li leg.; 1 spec., Napo, Beidou, 550 m, 12.iv.1998, Chunsheng Wu leg.; 3 spec., Shangsi, Hualan town, Hualan village, 204 m, in pool, 6.vii.2011, Keqing Song leg.; 1 spec., Hechi, 4.xi.1941, Zhelong Pu leg.; 4 spec., Jingxi, Bangliang, 6.viii.2010, Jianghua Huang leg. **Hainan**: 1 spec., Hainan, 16.XII.1957, Cuiying Li leg.; 1 spec., Xinglong, 24.xii.1957, Cuiying Li leg.; 1 spec., Yinggeling, 5.iv.2008, Yuxia Yang leg. **Yunnan**: 3 spec., Yingjiang, 25.v.1983, Lizhong Hua leg.; 1 spec., Jinping, Mengla, 500 m, 20.iv.1956, Keren Huang leg.; 1 spec., Pohui, 2.ix.1979, Zhelong Pu leg.; 1 male, Xishuangbanna Botanical Garden (west area), near Wanglian Hotel, 4–11.iv.2021, Huang Baoping leg.

#### Distribution.

China (Fujian, Guangdong, Guangxi, Hainan, Hong Kong, Hubei, Hunan, Jiangsu, Jiangxi, Shanghai, Sichuan, Yunnan, Zhejiang), Cambodia, Malaysia, Thailand, Vietnam. New for Hong Kong.

#### Habitat.

This species occurs in mud with aquatic grass at the edge of pool, or under root of waterside grass on the bank of slow stream. It can sometimes be collected by light trap.

### ﻿ Helochares anchoralis

Taxon classificationAnimaliaColeopteraHydrophilidae

Sharp, 1890

7A69DAC7-556E-5923-AB65-4B7CF15ACB20

Figs 37 , 44 , 63 , 64

Helochares anchoralis Sharp, 1890: 352. - Sri Lanka [Colombo].

#### Material examined.

**Jiangxi**: 1 female, Nanchang. **Guangdong**: 3 spec., Henan, 24.v.1957, Zhelong Pu leg.; 1 spec., Henan, Fenghuang, 25.xi.1957; 2 spec., Guangzhou, Lianxian, vi.1945, Zhelong Pu leg.; 2 spec., Dinghu, 10.v.1994, Fenglong Jia leg.; 2 sepc., Sun Yat-sen University campus, 15.iv.1958.; 1 spec., Guangzhou, viii.1938, Zhelong Pu leg.; 1 spec., Shaoguan, Yingde, 4.viii.1962, Ping Lin leg.; 1 spec., Shenzhen, Neilingding, 3.vii.1998, Haidong Chen leg.; 1 female, Shenzhen, Paiyashan, 17.v.2012, Fenglong Jia and Junlei Liao leg.; 1 spec., Zhuhai, Hengqin Island, 10.vii.2006, Fenglong Jia leg. **Guangxi**: 10 spec., Yangshuo, 1985, Shoujian Chen leg.; 7 spec., Guangxi, Nanning, 19.vi.1977, Zhihe Huang leg.; 2 spec., Nanning, vi.1958, Zhelong Pu leg. **Hainan**: 3 spec., Sanya, 24.xii.1963, Tongxu Peng leg.; 1 male, Lingshui, Diaoluoshan, 29.xii.1963, Zhenda Lin leg.; 1 female, Hainan, 19.XII.1963, Tongxu Peng leg. **Chongqing** : 1 spec., Chongqing, 8.iii.1942, Xiangzhi Chen leg. **Yunnan**: 3 spec., Jingdong, 1200m, 9.v.1957, A. Mengqiaciji leg.; 4 spec., Xiaomengyang, 850m, 4.v.1957, Qiuzhen Liang leg.; 1 female, Cheli, 500m, 7.iv.1955, Keleirangnuofusiji leg.; 1 female, Pohui, 2.ix.1939, Zhelong Pu leg.; 1 female, Jinping, Mengla, 370m, 22.iv.1956, Keren Huang leg.; 1 female, Hekou, 8.vii.1977, Zhihe Huang leg.

#### Distribution.

China (Chongqing, Fujian, Guangdong, Guangxi, Hainan, Hubei, Jiangxi, Sichuan, Taiwan, Yunnan), Bangladesh, Cambodia, India, Indonesia, Laos, Philippines, Sri Lanka, Thailand, Japan. New for Chongqing, Jiangxi and Guangxi.

#### Habitat.

This species occurs in mud with aquatic grass at the edge of pool. It can occasionally be collected by light trap.

## ﻿Discussion

**Figure 65.**
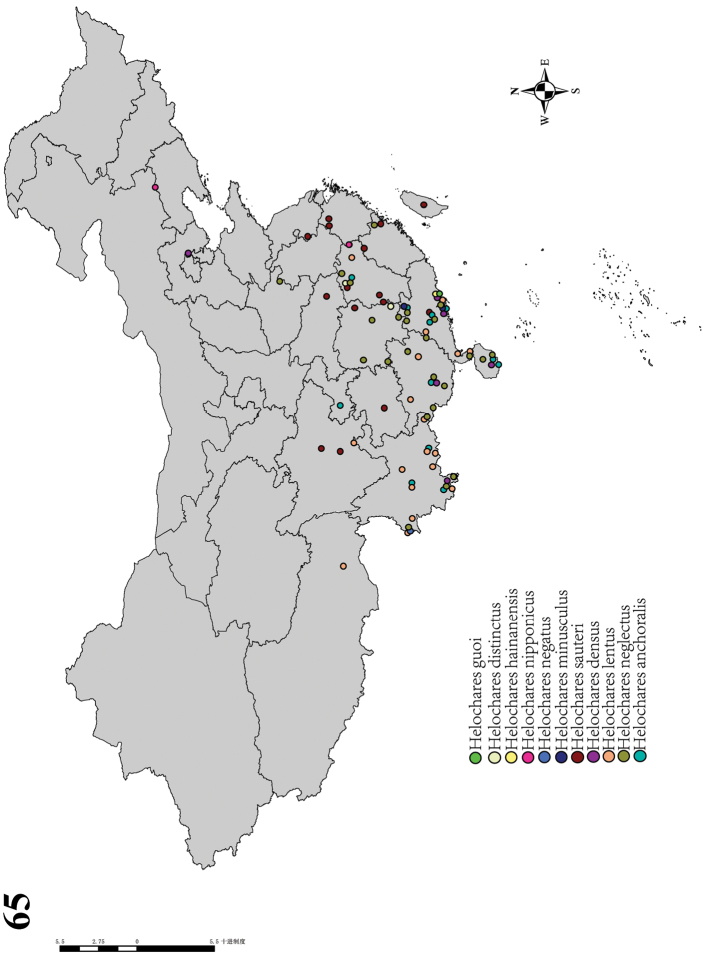
Distribution map of *Helochares* species in China.

*Helochares* is a typical tropical group that is mainly known from the Oriental and Afrotropical realms. Of the 20 species known from China, 18 occur south of the Qinling-Huaihe Line, *Helocharesobscurus* (Müller, 1776) occurs in Xinjiang and *H.nipponicus* Hebauer, 1995 in the Palearctic Realm from Jilin to Zhejiang (Fig. 65). It is very possible that *H.nipponicus* will be found in south China with further exploration.

Although over 160 species of *Helochares* are described in the world, it is likely that there is still enormous potential for more new species to be described in the Oriental Realm including the Chinese part. The discovery of new species and newly recorded species by Dong and Bian (2021) and us extends the known range of the genus in China. However, there are some dubious records.

d’Orchymont (1928) reported *H.crenatus* Régimbart, 1921 from Cambodia, Tokin, Bangladesh, Malaysia, Indonesia, Philippine, and India. d’Orchymont (1940) described *Helocharesnebridius* d’Orchymont, 1940 and identified all the material of *H.crenatus* from Indonesia (Java, Sumatra) as *H.nebridius*. He also provided a table in which distribution information of all known species of *Helochares* in the Oriental Realm was given. Based on this table, *H.crenatus* only occurs in “Inde continentale” and other records of this species were excluded (d’Orchymont, 1943a). The distribution of this species outside India comprises Thailand (Hebauer 1995) and China (Pu 1963). Pu (1963) first reported *H.crenatus* from Yunnan, China based on one specimen, which was followed by Gentili et al. (1995), Hansen (1999) and Dong and Bian (2021). There has been no other report of the species from China. The specimen Pu checked is deposited in IZCAS. The second author visited IZCAS in 2018 and checked the specimens from Yunnan, but unfortunately the specimen was a female. After studying the specimen, the second author did not find any difference with *H.neglectus* (Hope), a very common species in Yunnan. The specimen of *H.crenatus* checked by Pu is probably *H.neglectus* (Hope). So, the report of *H.crenatus* from Yunnan is dubious. We suggest removal of *H.crenatus* from the Chinese fauna.

Dong and Bian (2021) described *Helocharestengchongensis* Dong & Bian, 2021 from Yunnan. The species was compared with *Helochareslentus* Sharp, 1890, a species that is common in China. However, based on the original description and photos, *H.tengchongensis* is much closer to *H.densus* Sharp, 1890. Therefore, it is necessary to compare the types of *H.tengchongensis* and *H.densus* to shed light on the status of *H.tengchongensis*.

Dong and Bian (2021) described *Helochareswuzhifengensis* Dong & Bian, 2021 from Wuzhifeng town, Jiangxi Province, China. This species was originally compared with *Helocharesnipponicus* that is also distributed in eastern and northeastern China. We checked over 180 specimens from the Luoxiaoshan Mountain Range of which 147 specimens were from neighbouring areas of Wuzhifeng. All of the specimens we checked are *H.sauteri* d’Orchymont, 1943. Based on the photo of the aedeagus, *Helochareswuzhifengensis* is very similar to *H.sauteri* except for the apical process of the median lobe (see Dong and Bian, 2021: 170, fig. 7). We discussed with Bian the similarity between *H.sauteri* and *H.wuzhifengensis*. After carefully checking the holotype of *H.wuzhifengensis*, she told us that the aedeagus is very similar to that of *H.sauteri*, but the median lobe is much narrower apically (see Dong and Bian, 2021: fig. 7). However, the lateral membrane of the median lobe sometimes becomes nearly transparent after being treated with glacial acetic acid (Dong and Bian (2021) treated the aedeagus with this chemical). Therefore, we are not sure if the median lobes of the aedeagi of *H.wuzhifengensis* and *H.sauteri* are identical. This conflict may be solved by dyeing the aedeagus of the holotype of *H.wuzhifengensis*.

## Supplementary Material

XML Treatment for Helochares guoi

XML Treatment for Helochares distinctus

XML Treatment for Helochares hainanensis

XML Treatment for Helochares nipponicus

XML Treatment for Helochares negatus

XML Treatment for Helochares minusculus

XML Treatment for Helochares sauteri

XML Treatment for Helochares densus

XML Treatment for Helochares lentus

XML Treatment for Helochares neglectus

XML Treatment for Helochares anchoralis

## Supplementary materials

Supplementary material 1 Two new species of Hydrophilidae were described and additional faunastic records of *Helochares* from China were provided Data type species data https://binary.pensoft.net/file/624128This dataset is made available under the Open Database License (http://opendatacommons.org/licenses/odbl/1.0/). The Open Database License (ODbL) is a license agreement intended to allow users to freely share, modify, and use this Dataset while maintaining this same freedom for others, provided that the original source and author(s) are credited.Zhenming Yang, Fenglong Jia, Yudan Tang, Lu Jiang
