# Supplementary material for: ﻿Two new species of Helochares, with additional faunistic records from China (Coleoptera, Hydrophilidae, Acidocerinae)
Source: Zookeys. 2021 Dec 16;1078:57–83. doi: 10.3897/zookeys.1078.73458 (PMC8702532; doi:10.3897/zookeys.1078.73458)
Supplement: Supplementary material 1 — Two new species of Hydrophilidae were described and additional faunastic records of Helochares from China were provided [file zookeys-1078-057-s001.docx]

Two new species of Helochares (Coleoptera, Hydrophilidae) from China

Zhenming Yang, Fenglong Jia, Yudan Tang, Lu Jiang

Two new species, *Helochares* *guoi* Yang et Jia sp. nov. and *Helochares* *distinctus* Jia et Tang sp. nov. are described. Two species are recorded for the first time from China: *Helochares negatus* Hebauer, 1995 from Yunnan, and *Helochares* *minusculus* d'Orchymont, 1943 from Guangdong. Additional faunistic data from China are provided for the following species: *Helochares hainanensis* Dong et Bian, 2021, *Helochares nipponicus* Hebauer, 1995, *Helochares* *sauteri* d'Orchymont, 1943, *Helochares densus* Sharp, 1890, *Helochares* *lentus* Sharp, 1890, *Helochares neglectus* (Hope, 1854), *Helochares* *anchoralis* Sharp, 1890. All recorded species are illustrated.
